# Auditory processing in rodent models of autism: a systematic review

**DOI:** 10.1186/s11689-022-09458-6

**Published:** 2022-08-30

**Authors:** Maya Wilde, Lena Constantin, Peter R. Thorne, Johanna M. Montgomery, Ethan K. Scott, Juliette E. Cheyne

**Affiliations:** 1 The Queensland Brain Institute, The University of Queensland, Brisbane, QLD 4072 Australia; 2 Department of Physiology, Faculty of Medical and Health Sciences, Centre for Brain Research, University of Auckland, Auckland, New Zealand; 3 Section of Audiology, School of Population Health, Faculty of Medical and Health Sciences, University of Auckland, Auckland, New Zealand; 4 Department of Anatomy and Physiology, School of Biomedical Sciences, The University of Melbourne, Parkville, VIC 3010 Australia

**Keywords:** Autism spectrum disorder, Auditory, Rodent models, Auditory brainstem recordings, Cortical event-related potentials

## Abstract

Autism is a complex condition with many traits, including differences in auditory sensitivity. Studies in human autism are plagued by the difficulty of controlling for aetiology, whereas studies in individual rodent models cannot represent the full spectrum of human autism. This systematic review compares results in auditory studies across a wide range of established rodent models of autism to mimic the wide range of aetiologies in the human population. A search was conducted in the PubMed and Web of Science databases to find primary research articles in mouse or rat models of autism which investigate central auditory processing. A total of 88 studies were included. These used non-invasive measures of auditory function, such as auditory brainstem response recordings, cortical event-related potentials, electroencephalography, and behavioural tests, which are translatable to human studies. They also included invasive measures, such as electrophysiology and histology, which shed insight on the origins of the phenotypes found in the non-invasive studies. The most consistent results across these studies were increased latency of the N1 peak of event-related potentials, decreased power and coherence of gamma activity in the auditory cortex, and increased auditory startle responses to high sound levels. Invasive studies indicated loss of subcortical inhibitory neurons, hyperactivity in the lateral superior olive and auditory thalamus, and reduced specificity of responses in the auditory cortex. This review compares the auditory phenotypes across rodent models and highlights those that mimic findings in human studies, providing a framework and avenues for future studies to inform understanding of the auditory system in autism.

**Supplementary Information:**

The online version contains supplementary material available at 10.1186/s11689-022-09458-6.

## Introduction

### Background

Autism, or autism spectrum disorders (ASD), refers to a wide range of developmental conditions that affect at least one in every 100 people worldwide [66]. As well as complex and heterogenous traits, the aetiology of autism comprises a combination of hundreds of genetic and environmental factors, with many likely still unknown [46, 63, 134]. The primary diagnostic symptoms of ASD are difficulties with social interaction and verbal communication, and repetitive behaviours, which may include restricted interests [22]. While differences in sensory perception have long been recognised by the autism community, sensory sensitivity was not added to the diagnostic criteria until 2013, and research into this facet of autism has greatly increased in recent years [3, 112]. The vast majority of people with autism present with hypersensitivity or (less commonly) hyposensitivity of sensory modalities such as hearing, vision, and touch [19, 76, 129, 139]. Difficulty and delays with spoken language are also common and may result either from differences in attention or auditory processing [26, 76, 112, 139]. These phenotypes may arise from higher structures, such as the auditory and prefrontal cortices, or subcortical structures that pass along heightened auditory responses to the cortex [77].

Measures of activity in the human auditory system, such as electroencephalography (EEG), magnetoencephalography (MEG), functional magnetic resonance imaging (fMRI), and auditory evoked responses (including auditory brainstem responses (ABRs) and cortical event-related potentials (ERPs)) have provided some level of understanding of the underlying bases of auditory phenotypes in autism (reviewed by [76]). These are all non-invasive methods of recording brain activity, with limited spatial resolution: they measure average activity in a brain region and cannot give information about cellular-level activity. Rodent studies have the benefit of pairing these non-invasive methods with cellular techniques including electrophysiology and histology, for more spatially precise understanding of structural and functional relationships in the auditory system. Studies of auditory processing in humans with autism often provide conflicting results in whether differences in auditory processing exist between cohorts of people with and without autism, and the direction and magnitude of those differences [131, 144]. These conflicts may arise from the difficulty of controlling for autism aetiology in human studies: autism arises from a wide range of genetic and non-genetic factors, and different aetiologies do not necessarily share the same phenotype [131, 137]. This, in turn, can dictate the intensity of autistic traits in a person or in an animal model, including auditory processing. Some studies control for aetiology by recruiting only those with a syndromic form of autism, such as fragile X syndrome (FXS) or Rett syndrome, rather than idiopathic ASD (iASD) [137]. In rodent studies, the aetiology is determined by the experimental model, and it is, therefore, important to compare across a range of models to form an impression of iASD as a whole. This review aims to characterise the function of structures along the ascending auditory pathway in a wide range of rodent models of ASD by comparing results from histology, ABR, electrophysiology, EEG, fMRI, and behavioural tests. Comparing across these models will enable us to compare auditory phenotypes between different genetic and environmental factors in highly controlled groups, which is not possible in human populations. Conclusions from these comparisons will aid understanding of how auditory information is processed differently in autism compared to in typical development.

### Anatomy of the ascending auditory processing pathway

**Fig. 1:**
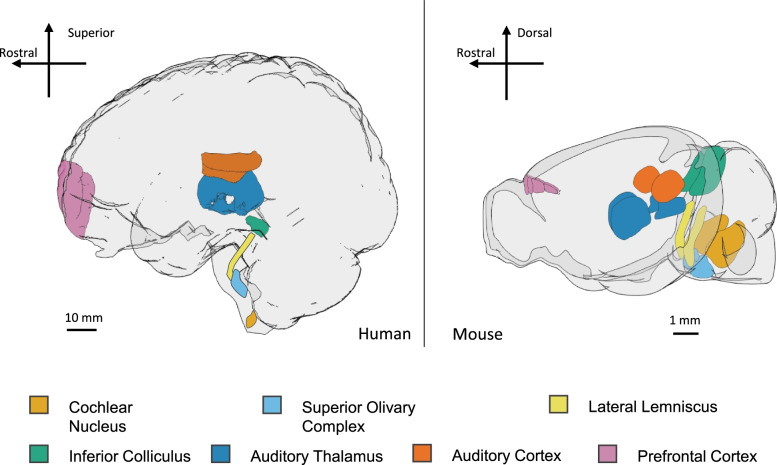
Key structures in the auditory processing pathway in the human and mouse brain. Auditory information enters the central nervous system at the cochlear nuclei and is then processed at the level of the superior olivary complex, lateral lemniscus, inferior colliculus, auditory thalamus (medial geniculate nucleus and thalamic reticular nucleus), auditory cortex, and prefrontal cortex. Scale bars are approximate. Images generated with brainrender [18]

The key structures in the ascending auditory pathway are largely conserved between humans and rodents, though naturally at different scales (Fig. 1). The cochlear nuclei are the first structures in the central auditory pathway, receiving input from the cochlea via the cochlear nerve. The cochlear nuclei consist of the dorsal cochlear nucleus (DCN) and the ventral cochlear nucleus (VCN). The latter can be further divided into the anterior VCN (AVCN) and posterior VCN (PVCN), based on these two areas containing different cell types [51]. After the cochlear nuclei, an important feature of this pathway is its bilaterality: information is passed both ipsilaterally and contralaterally along the pathway for comparison of features of the sound inputs to the left and right ears. The VCN projects mostly ipsilaterally to the superior olivary complex (SOC). Key structures in the SOC are the medial superior olive (MSO), lateral superior olive (LSO), medial nucleus of the trapezoid body (MNTB), lateral nucleus of the trapezoid (LNTB), and the superior paraolivary nucleus (SPON) [51, 72]. The MSO and LSO receive excitatory input from the VCN and inhibitory input from the MNTB. The LNTB also provides inhibitory input to the MSO. The lateral lemniscus (LL) comprises a dorsal and ventral nucleus (DNLL and VNLL) and relays information from the cochlear nuclei and the SOC onward to the inferior colliculus (IC) [98]. The central nucleus of the inferior colliculus (CNIC) receives direct excitatory input from the ipsilateral DCN, as well as the contralateral VCN and CNIC, and inhibitory input from the VNLL and the LSO. It then passes excitatory input along to the medial geniculate nucleus (MGN) of the thalamus. The two major auditory structures of the thalamus are the MGN and the thalamic reticular nucleus (TRN). These integrate auditory information from the CNIC and pass it on to the cortex [9]. The primary location for the processing of auditory stimuli once signals reach the cortex is the auditory cortex, which is composed of several distinct areas but most notably the primary auditory cortex (A1). In rodents, other fields include the secondary auditory cortex (A2) and anterior auditory field (AAF). In primates, the auditory fields include the core (primary), belt (secondary), and parabelt (tertiary) [120]. The prefrontal cortex (PFC) is then involved in interpreting auditory information and producing appropriate responses to complex sounds. As auditory information is passed through this pathway, information such as rapidness, location, frequency, and intensity of sound is relayed through spatial and temporal properties of these structures. For example, neurons in structures along the rodent auditory processing pathway typically show frequency tuning: a preference in their responses for a specific ‘characteristic frequency’, with much weaker responses the further removed a sound is from that frequency [56]. Tonotopic arrangement of neurons within these structures generally places those which preferentially respond to low-frequency sounds at one end of the structure and those which preferentially respond to high-frequency sounds at the opposite end, although there can be some variation in this across species [12, 120]. While the scale of these structures, the hearing range, and the capacity for higher-order functions (such as understanding spoken language) are not conserved between rodents and humans, basic properties such as frequency specificity and transmission of activity between the auditory structures are sufficiently well conserved between human and rodent brains to warrant comparisons between findings in rodent models and human cohorts [125].

### Rodent models of autism

A wide range of genetic and teratogenic factors involved in autism have been identified, though these still do not account for all individuals with autism [25, 46, 63]. There are now over 200 rodent models bearing genetic mutations in genes that have been identified as having variants in people with autism, and over 40 produced by maternal exposure to teratogens [1, 10, 34]. It is worth noting, however, that the same genetic variant may produce variable effects in people with autism and that many people likely develop autism as a result of interactions between multiple genetic variants and/or non-genetic factors [134]. Given that rodent models are typically focused on a single mutation or teratogenic exposure, they do not necessarily model human autism accurately [22]. For this reason, autism research often focuses on endophenotypes (measures of activity that underlie broader behavioural phenotypes) that may arise similarly from different genetic backgrounds [54]. As such, identifying endophenotypes in rodent models with a single genetic mutation or teratogenic exposure may be generalisable to humans exhibiting similar endophenotypes even though the human aetiology may be more complex [43, 83].

Rodent models of autism are typically validated by assessing social, repetitive, and anxiety behaviours that may have similarities to humans. However, a direct comparison to the clinical diagnosis of autism is not straight-forward, as this is a more complex process. Relevant to this review, sensory phenotypes are now more likely to be included in the autism assessment in humans and thus recent studies have called for their inclusion in the validation of rodent models [125, 128]. Proxies in rodent studies, for sensory experiences that would be measured by questionnaire in humans, include behavioural responses and recordings of evoked neural activity, which can indicate hypersensitivity or hyposensitivity [125].

Rodent models of autism include those with mutations in genes encoding a range of different proteins [1]. Some of these, such as *Fmr1* and *Mecp2*, are associated with their own syndromes (FXS and Rett syndrome, respectively), which have high co-occurrence with autism and are therefore included as models of ASD [103, 137]. Unsurprisingly, given the neurodevelopmental nature of autism, many of these genes have especially important roles during the development of the nervous system. Some cellular functions fulfilled by proteins encoded by autism-associated genes include intracellular Ca^2+^ signalling, synaptic transmission, and chromatin remodelling [100, 134]. Furthermore, several of these proteins are ‘master regulators’ of genetic expression: through their roles interacting with DNA, RNA, or proteins, they directly affect the expression or function of other autism-related genes and proteins [100]. Mutations affecting the function of these proteins therefore have more widespread effects across a group of genes which can also independently have mutations that link to autism. Other genetic rodent models incorporate microdeletions: segments of chromosome containing several genes which are deleted in order to model similar deletions found in people with autism [23, 39, 57].

Non-genetic rodent autism models use teratogens such as valproic acid (VPA), thalidomide, and lipopolysaccharide (LPS) to produce an autism-like phenotype [4, 38, 53]. These teratogens have been found to increase the likelihood of autism in children when present prenatally, and rodent models may receive the drug either once or multiple times during gestation, or even postnatally to mimic the later stages of human pregnancy [25, 131].

**Fig. 2:**
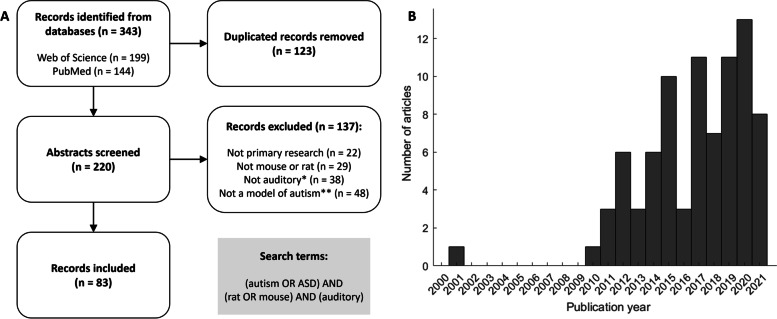
**A** Search strategy for systematic review. Records were found from searches in two databases. After removing duplicated entries, 234 abstracts were screened for inclusion in the review. Of these, 88 were primary research articles investigating the structure and function of the auditory processing pathway in mouse or rat models of autism. **B** Publication year of the records which were included in the review. Almost all records were published in the last 10 years

This review focuses on auditory phenotypes in autism and draws together recent studies that have described auditory function in rodent models of ASD. Some models, such as *Fmr1* and VPA, have been used in a high number of studies compared with other models (Supplementary Table 2). This is likely due to their robust phenotypes in other areas of ASD research and conclusive status as an aetiology of autism in humans. However, over recent years, there has been an increase in publications which study auditory function in a wider range of models (Fig. 2B). It is particularly important that models outside of *Fmr1* be investigated in auditory studies, as human studies have found that people with FXS often have different auditory phenotypes to those with iASD [131]. By assessing the function of the auditory system across many studies in different ASD models, a more detailed understanding of changes in the auditory network can be achieved.

In summary, the purpose of this systematic review is to bring together findings in the central auditory function of many rodent models of autism, in order to find common auditory endophenotypes. Additionally, we aim to summarise the results of invasive studies in these models, which may provide explanations for the underpinnings of these endophenotypes. By comparing across these models, we aim to identify which results are common between autism models and which are specific to certain aetiologies. By comparing similarities and differences between models with controlled aetiologies, we hope to shed light on the often-conflicting results of human studies, which usually comprise a cohort of people with mixed or unknown aetiologies (with the exception of syndromic studies).

## Methods for the systematic review

This systematic review was conducted in accordance with the PRISMA guidelines [94]. Searches for primary research articles were conducted using two databases: Web of Science (Clarivate) and PubMed (Elsevier), on the 2nd of May, 2022. In both search engines, the search keywords were (‘autism’ OR ‘ASD’) AND (‘rat’ OR ‘mouse’) AND (‘auditory’). These two searches yielded a total of 362 results, 128 of which were either duplicated within a search or between the two search engines (Fig. 2A). There were no entries that were inaccessible or not in English. The remaining 234 unique abstracts were screened for inclusion as primary research articles in this review. Abstracts were included in the final list according to four criteria: primary research articles, experiments were performed in either mice or rats, focus included central auditory processing, and that an appropriate model of autism was examined. Entries including reviews and conference abstracts and those studying humans or other animals and not mice or rats were excluded. Entries were included if the study investigated structure or function in the ascending central auditory processing pathway. Paradigms such as auditory fear conditioning and ultrasonic vocalisations were excluded as they are designed to test fear learning and communication, rather than auditory function. A study which only investigated peripheral hearing in a mouse model of autism was also not included [16]. Careful consideration was paid to inclusion of studies on the basis of whether the model in question represented a robust model of autism. For example, in the case of genetic models, studies were included if the gene in question is included on the SFARI gene website as category 1 (high confidence), 2 (strong candidate), or 3 (suggestive evidence). Models were included whether they were full knockouts of the gene, heterozygous knockouts, or conditional knockouts specifically in certain cell types or for limited periods. In the case of *Mecp2*, both over- and under-expression of the gene are associated with autism, and models of both were included. Models with chromosomal microdeletions were also included if they have several reports listed in the SFARI gene database [1]. Autism-related genes affected by these deletions can be found in Supplementary Table 2. For teratogenic models, these were included if they were listed in the SFARI induced model database with strong human clinical evidence associated. A full list of the 88 included primary research articles can be found in Supplementary Table 1. Altogether 36 genes, microdeletions and teratogens are modelled across these articles (Supplementary Table 2). Multiple models were used in some studies, and in these cases, only the findings from the models which fit the inclusion criteria were included. Our search does not include articles which assess auditory function in models of syndromes such as FXS or Rett syndrome if they did not also identify their model as linked to autism. Our search may also have missed articles which did not use ‘auditory’ as a keyword, though ‘auditory’ was used to find studies of central rather than peripheral processing (such as may have been found by the keyword ‘hearing’).

The findings of these 88 studies relevant to central auditory processing are divided below into non-invasive measures with human counterparts which are candidates for auditory endophenotypes and invasive measures which may explain the cellular origins of these endophenotypes.

## Results

The models referred to in this review encompass a range of mouse and rat strains. Unfortunately, some of the studies included use models on a background of the C57BL/6 mouse strain, which is known to be associated with a peripheral, progressive high-frequency sensorineural hearing loss from 3 months of age, and is even used as a model of early onset hearing loss [95]. Auditory experiments conducted in these animals therefore have an underlying caveat affecting wild type (WT) hearing, which may make it difficult to parse out differences in audition caused by the autism model versus those resulting from the strain’s genetic background. There were 17 studies that used this mouse strain (above 3 months of age) and this is noted throughout this ‘Results’ section, as well as in Supplementary Table 1.

### Non-invasive measures of auditory function

#### Auditory brainstem responses

**Fig. 3:**
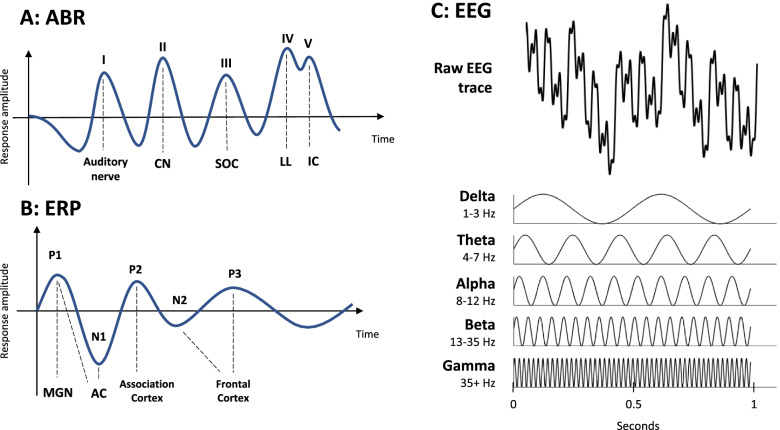
Measures of function in along the auditory pathway. **A** Example human auditory brainstem response (ABR) trace. The first peak represents activity in the auditory nerve, and the second correlates to the cochlear nucleus. Peak III represents the superior olivary complex, and peaks IV and V represent the lateral lemniscus and the inferior colliculus, respectively. **B** Example cortical auditory event-related potential (ERP) trace. The ERP is made up of 5 peaks, representing activation in different areas. P1 is produced by activity in the auditory thalamus (MGN) and the primary auditory cortex, N1 is produced by the auditory cortex. P2 is produced by the association cortex. N2 and P3 peaks (associated with the frontal cortex) are observed in humans, but less commonly in rodents. **C** Exemplar EEG frequency activity. Raw EEG traces (as may be recorded from the auditory or prefrontal cortices) are composite waves, from which activity in several frequency ranges can be extracted. The power in each of these frequency bands indicates the extent to which activity at that frequency contributes to the overall recorded activity

Auditory brainstem response (ABR) recordings are non-invasive measures used clinically by audiologists to assess the transmission of information through the subcortical auditory pathways and intensity thresholds for responses to sound [51]. Equivalent recordings to those in humans of all ages can also be made in rodents, where they are often used to measure auditory function, particularly auditory thresholds (sensitivity). The output trace has several peaks, which correspond to the activity in subsequent brain regions along the auditory pathway in response to a sound stimulus (Fig. 3A). In children with autism, the most common findings from ABR recordings are increased latencies, especially of peaks III and V (representing delayed activation of the SOC and IC), although this effect is reduced or even reversed in adulthood [82, 99, 126]. Findings of changes to the amplitude of ABR peaks (which would correspond to more or less activity in the relevant brain area) in autism are much less conclusive [99, 126]. Results of ABR studies in rodent models of ASD are summarised in Table 1. The majority of studies show no difference in ABR thresholds compared to WTs, although half of these studies were conducted in C57BL/6 mice over 3 months old [37, 64, 71, 89, 124, 152]. However, *Fmr1*^*−/−*^ and *Adnp*^*+/−*^ mice have higher thresholds than WTs, indicating less sensitivity to quieter sounds [50, 116]. A study in 16p11.2 microdeletion mice also used ABRs to test hearing thresholds, but found no responses and concluded that this mutation caused deafness in these mice [151].

**Table 1:** Auditory brainstem responses in rodent models of ASD. Model names are written in bold if the result represents more than one study, or the majority of studies in the case where results from a single model are mixed. Numbers in subscript brackets refer to the relevant papers from the literature search, as numbered in Supplementary Table 1. Models are listed in alphabetical order

Measure	Models increased compared to WT	Models unchanged compared to WT	Models decreased compared to WT
**ABR threshold**	*Adnp*_(28)_, *Fmr1*_(67)_	*Chrna7*_(18)_, *Cntnap2*_(70)_*, Drd2*_(42)_, ***Mecp2***_(35, 86)_, *Wnt1*_(52)_	*Wnt1*_(52)_
**Peak I Amplitude**		*Cntnap2*_(70, 71)_, *Drd2*_(42)_	
**Peak II Amplitude**	*Cntnap2*_(70)_	*Drd2*_(42)_, *Fmr1*_(67)_	
**Peak III Amplitude**	*Cntnap2*_(70)_, *Fmr1*_(67)_	*Cntnap2*_(70,71)_, *Drd2*_(42)_	
**Peak IV Amplitude**	*Cntnap2*_(70)_	*Cntnap2*_(70,71)_	
**Peak I Latency**		*Cntnap2*_(70,71)_, *Drd2*_(42)_	
**Peak II Latency**		*Drd2*_(42)_, *Fmr1*_(67)_	
**Peak III Latency**	*Cntnap2*_(70)_	*Drd2*_(42)_, *Mecp2*_(86)_	
**Peak IV Latency**		*Cntnap2*_(71)_	*Cntnap2*_(70)_

Changes in the amplitude or latency of the ABR peaks in autism models would indicate changes in the intensity or delay of the response in certain auditory structures. However, studies of the amplitude and latency of ABR peaks in autism models are sparse and have inconclusive results (Table 1). Most studies show no difference to WTs, and in several cases where there are differences at young ages, these shift to resemble WTs in older animals [71, 116, 124]. Differences in ABR latencies are also most evident in humans at young ages, and it has been recommended that rodent studies examine a range of ages in order to capture such developmental changes between juveniles and adults [128]. Most studies using ABRs have found few and varied differences between WTs and ASD model animals, and there are few coherent patterns across studies within and across rodent models. As appealing as ABRs are as a non-invasive measure of function in lower auditory structures, too few studies have performed this test on rodent models of ASD to draw conclusions across different models. ABR measures in children with autism show trends towards lower amplitudes and longer latencies, but there is a significant variation in the response [74, 76, 136]. The inability of rodent studies in various models to converge on a consistent ABR phenotype may therefore be an accurate portrayal of the diversity of ABRs in iASD with a range of aetiologies.

#### Cortical event-related potentials

EEG is a useful non-invasive tool for measuring cortical activity in humans, both at rest and in response to stimuli. In rodents, EEG generally uses implanted electrodes, and typically just one to three sites, though more recent studies have used up to 30 channels [55]. These methods can be used to measure the cortical auditory event potentials that follow ABRs in response to sound stimuli (Fig. 3B). As the included studies refer to these waveforms simply as auditory event-related potentials (ERPs), that term will be used in this review. These ERPs have a distinct shape, including well-defined positive peaks (P1, P2, and P3) and negative peaks (N1 and N2), which are associated with different aspects of sound processing [131, 148]. The P1 is produced by a combination of activity in the auditory thalamus and the primary auditory cortex, the N1 by the auditory cortex, and the P2 by the association cortex [59, 83, 148]. The N2 and P3 peaks (associated with the frontal cortex) represent higher-order activity and are observed in humans, but appeared in very few of the studies included in this review. The N1 peak has been shown to be reduced in amplitude in people with autism, but increased in amplitude in those with FXS [35, 83, 107, 131]. These changes represent reduced evoked activity in the auditory cortex in iASD, but stronger activity in the auditory cortex in FXS. The N1 amplitude is therefore a prime example in which auditory phenotype is certainly not generalisable across aetiologies. In Rett syndrome, the amplitude of N1, P2, and N2 may be decreased, and latencies across most peaks tend to be increased [119, 136]. In people with iASD, the most consistent difference in the ERP profile is increased latency of the N1 peak, indicating slower transmission of information to the auditory cortex [43, 105]. It is known that ERPs change with age in humans and rodents, notably that the N1 and P2 amplitudes are lower, and P1 and N1 latencies are increased in children and young mice compared to adults [131]. The reduced N1 amplitude and increased N1 latency seen in people with autism may therefore represent delayed maturation of auditory circuits.

A summary of analyses of auditory event-related potentials in rodent models of ASD is shown in Table 2. In cases where more than one study examined the same model, the most common result is indicated by bold font. It should be noted that a subset of these studies measured ERPs in anaesthetised animals [28, 30, 32, 60, 70, 123]. Anaesthesia has been shown to disrupt auditory and other sensory event-related potentials [5, 114]. Results in Table 2 which come solely from studies in anaesthetised animals are therefore indicated with an asterisk. It is noted that these studies are largely in rats: only two *Fmr1*^*−/−*^ mouse studies used anaesthesia, and the results from these merged with those from awake *Fmr1*^*−/−*^ mice. The majority of studies used white noise as a stimulus, though some used recorded human speech sounds or pure tones [28–30, 32, 60]. Human studies typically use simple stimuli such as pure tones, but it has been suggested that more complex sound stimuli can be informative and should also be used in rodent studies for translational validity [83, 125]. Furthermore, most studies do not compare a range of stimulus presentation rates to generate average ERP traces. One study that did so found the habituation rate of the N1 amplitude was lower in *Fmr1*^*−/−*^ mice, but only at presentation rates greater than one stimulus per second [70]. This difference in habituation could lead to detection of differences in N1 amplitude in the mean trace.

**Table 2:** Auditory event-related potentials in rodent models of ASD. Entries are in bold if they are supported by more than one study or represent the result of the majority of the studies using that model (if there is no majority, no result is in bold). Numbers in subscript brackets refer to the relevant papers from the literature search, as numbered in Supplementary Table 1. Asterisks indicate cases where all of the studies contributing to that result were conducted in anaesthetised animals. Models are listed in alphabetical order

Measure	Models increased compared to WT	Models unchanged compared to WT	Models decreased compared to WT
**P1 amplitude**	*Fmr1*_(30)_	***Fmr1***_(32, 37, 73, 83)_, ***Mecp2***_(26, 35)_, *NR1*_(69)_	15q13.3_(31)_
**N1 amplitude**	Cntnap2*_(71)_, ***Fmr1***_(30, 32, 37, 83)_, ***Mecp2***_(16, 26, 35)_, *MGluR5*_(4)_, VPA*_(13)_	15q13.3_(31)_, *Fmr1*_(37, 83)_, *NR1*_(69)_, *Pcdh10*_(59)_	*Ehmt1*_(9)_, Fmr1*_(14)_, VPA*_(13)_
**P2 amplitude**	Cntnap2*_(71)_, *Fmr1*_(30)_, *Mecp2*_(26)_	***Fmr1***_(30, 32, 37, 83)_, *Mecp2*_(26, 35)_	Fmr1*_(14)_, Mecp2*_(16)_, *MGluR5*_(4)_, VPA*_(13)_
**P1 latency**	*Fmr1*_(37)_, *Mecp2*_(26)_	***Fmr1***_(30, 32, 83)_, *Mecp2*_(26, 35)_, *NR1*_(69)_	
**N1 latency**	Cntnap2*_(71)_, *Fmr1*_(37, 83)_, ***Mecp2***_(16, 26)_, ***NR1***_(22, 69)_, VPA_(23)_	***Fmr1***_(30, 32, 37, 40, 83)_, *Mecp2*_(35)_, *Pcdh10*_(59)_	
**P2 latency**	Cntnap2*_(71)_, *Fmr1*_(30)_, ***Mecp2***_(16, 26, 35)_	*Mecp2*_(26)_, *Fmr1*_(30, 37, 83)_	

##### P1

Many studies with *Fmr1*^*−/−*^ mice have found no difference in the amplitude of the P1 peak, though a recent study using a higher number of channels found an increased P1 in *Fmr1*^*−/−*^ mice compared to WTs [55, 60, 67, 130, 147]. The P1 amplitude is generally not different to WTs in other ASD models, with the exception of 15q13.3 deletion mice [48, 57, 64, 121].

##### N1

In *Fmr1*^*−/−*^ mice, the amplitude of N1 is usually increased compared to WTs, more commonly when measured from the frontal rather than the auditory cortex [55, 60, 67, 70, 130, 147]. However, N1 amplitude is decreased across the auditory cortex in anaesthetised *Fmr1*^*−/−*^ rats [30]. The increase seen in the mice resembles the auditory endophenotype of people with FXS. The amplitude of the N1 peak in other models is variously increased, decreased, or unchanged [8, 20, 28, 32, 48, 57, 64, 106, 121, 123]. Some of the increased and unchanged N1 amplitude results [8, 57, 64, 106] come from studies in C57BL/6 mice over 3 months old, which may impede their ability to accurately portray an auditory endophenotype. Nevertheless, it appears that the consistently reduced N1 amplitude in people with iASD and Rett syndrome is not well recapitulated across a wide range of rodent models.

##### P2

Mixed results are also visible in the amplitude of the P2 peak, with awake mice again mostly showing increased or unchanged amplitudes [48, 55, 60, 64, 67, 147]. Anaesthetised rats and *mGluR5*^*−/− interneuron*^ mice on a C57BL/6 background have decreased P2 amplitudes, with the exception of *Cntnap2*^*−/−*^ rats [8, 28, 30, 31, 123]. N2 and P3 amplitudes are not included in Table 2 as few studies reported them, but they were found to be decreased in anaesthetised rat *Fmr1*^*−/−*^ and *Mecp2*^*+/−*^ models, similarly to P2 [30, 32].

**Fig. 4:**
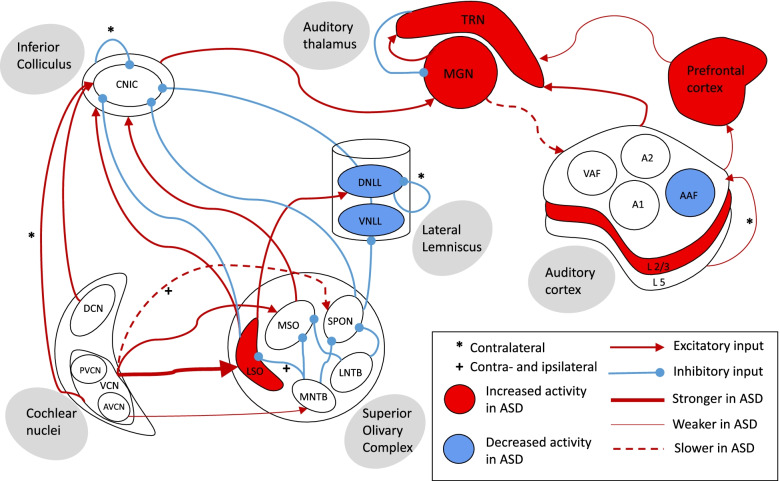
The ascending auditory processing pathway with presumed changes in autism based on results from studies in rodent models. Excitatory (red) and inhibitory (blue) connections between structures along the pathway are illustrated along with the changes to these connections and the activity within areas in rodent models of autism. Connections are primarily ipsilateral unless otherwise stated. There is increased activity in structures such as the lateral superior olive (LSO), the medial geniculate nucleus of the thalamus (MGN), thalamic reticular nucleus (TRN), the auditory cortex (specifically in layers 2/3), and the prefrontal cortex. Activity is consistently decreased in the dorsal and ventral lateral lemniscus (DNLL and VNLL) and the anterior auditory field (AAF) of the rat auditory cortex. Activity in other areas is either unchanged or results are conflicted. The connection from the ventral cochlear nucleus (VCN) to the LSO is increased in strength, while the strength of the connection from the anterior VCN (AVCN) to the medial nucleus of the trapezoid body (MNTB) is decreased. Signals from the VCN take longer to reach the SPON and from the MGN take longer to reach the auditory cortex in rodent models of autism. The speed of other connections is either unchanged or disputed between studies. The strength of connections between contralateral auditory cortices and between the auditory and prefrontal cortex is decreased, as is the feedback connection from the prefrontal cortex to the TRN

Whether latencies of ERP peaks differ from WTs in autism rodent models varies between studies. When differences are observed, they take the form of extended latencies in the ASD models, indicating slower transmission of auditory information [32, 42, 43, 48, 55, 60, 64, 67, 70, 106, 121, 147]. For several models, N1 latency is consistently increased (Table 2), which is very promising as it reflects one of the most consistent ERP endophenotypes in iASD [43, 105]. Note the N1 latency is less often increased in *Fmr1*^*−/−*^ animals, and increased N1 latency is not generally observed in people with FXS [131]. This result is represented in Fig. 4 as decreased speed of transmission from the auditory thalamus to the auditory cortex in iASD. In some studies that measured ERPs across several ages, differences in ERP peak latencies were not apparent in mice under 2 months old, but increased compared to WT at 2–3 months of age [48, 147]. This may represent a developmental phenotype that arises due to delayed maturation of the auditory circuit (i.e. the ASD models maintain an ‘immature’ ERP).

It has been proposed that ERPs could serve as a biomarker for differences in auditory processing in autism [83, 119]. In particular for people with iASD and Rett syndrome, the decreased amplitude and increased latency of various peaks, but especially the N1, seem to be the most consistent feature [119, 131, 136]. Across the studies included in this review, increased N1 latency appears to be recapitulated well in iASD and *Mecp2* rodent models, but decreased amplitude of N1 is poorly conserved. In people with FXS, the most consistent ERP phenotype is increased amplitude of the N1 [107]. This appears to be well recapitulated in *Fmr1* mouse models. Altogether, ERPs remain a promising cross-species auditory endophenotype, though further work is needed for robust recapitulation of the decreased N1 amplitude.

#### EEG power spectrum

Ongoing activity from EEG recordings can be separated out into activity occurring at different frequencies (Fig. 3C). Activity in each of these ranges correlates to connectivity across different parts of the brain: slower waves (e.g. delta) are produced by longer-range connections, and faster waves (e.g. gamma) are produced by activity within a cortical area [83]. This activity can be measured in auditory and frontal cortices, both at rest and following sound stimuli. In people with autism, there is evidence for a u-shaped change in resting frequency power, with an increase in the power at lower frequencies (delta and theta), decrease in middle frequencies (alpha), and increase at the highest frequencies (beta and gamma) of cortical activity [144]. However, following auditory stimulation, there is unchanged or even decreased evoked gamma power in ASD [43, 104, 105]. In Rett syndrome, the u-shape in resting EEG also appears to be present, only without the increase in gamma activity [113]. People with FXS show increased gamma power both at rest and evoked by sound stimuli, although in some instances the relative increase at baseline limits the ability to further increase in response to sound [35, 36, 107, 122]. A more consistent gamma activity EEG phenotype in people with ASD and FXS appears to be a decrease in inter-trial coherence (ITC) of gamma frequency activity, which represents disruption to local functional connectivity within the auditory cortex [35, 36, 43, 83, 103–105, 107]. It has been argued, however, that ITC values may not be as informative as they seem [142].

A summary of EEG activity in mouse models of autism is presented in Table 3. The most consistent results across studies are in the gamma frequency range. Resting gamma power is increased across a range of models in repeated studies [44, 48, 55, 67–69, 101, 102, 130, 147]. The few studies which showed no change in resting gamma power were all conducted in C57BL/6 mice over 3 months of age [8, 57, 106]. This matches the human endophenotype for increased resting gamma activity in the auditory cortex of people with autism and FXS, though this finding in *Mecp2* animals was surprising given the lack of resting gamma differences in children with Rett syndrome [36, 113, 144]. Evoked gamma power is almost always increased in *Fmr1*^*−/−*^ mice and decreased in all other ASD models [20, 43, 44, 48, 57, 67–69, 106, 147]. The few studies that diverge from this trend were in *mGluR5*^*−/− interneuron*^ and *Mecp2*^*+/−*^ mice on a C57BL/6 background [8, 49, 64]. Again, this strongly matches the divergent endophenotypes of FXS and iASD in humans. ITC of gamma activity is decreased in the auditory and frontal cortices of many ASD models [20, 42, 43, 48, 55, 57, 68, 101, 102]. Fewer studies found increased or unchanged gamma ITC, and most of these were in C57BL/6 mice over 3 months old [49, 64, 106]. This is another promising replication of one of the more consistent EEG phenotypes in both FXS and iASD.

**Table 3:** Summary of EEG activity power in different frequency spectra. Entries are in bold font in cases where a result is represented by more than one study in a model or represents the majority of the studies in that model. Numbers in subscript brackets refer to the relevant papers from the literature search, as numbered in Supplementary Table 1. Models are listed in alphabetical order

Measure	Models increased compared to WT	Models unchanged compared to WT	Models decreased compared to WT
**Resting delta**	***Fmr1***_(30, 37, 38)_	*Fmr1*_(30, 39, 58)_	
**Resting theta**	*Fmr1*_(30, 38)_	*Fmr1*_(30, 39, 58)_, *MGluR5*_(4)_	
**Resting alpha**	*Fmr1*_(30, 57)_	***Fmr1***_(30, 38, 39, 58)_, *MGluR5*_(4)_, *Pcdh10*_(59)_	
**Resting beta**	*Fmr1*_(30, 38)_	*Fmr1*_(39, 58)_, *MGluR5*_(4)_, *Pcdh10*_(59)_	
**Resting gamma**	***Fmr1***_(30, 37, 38, 39, 57, 58, 73, 83)_, *Mecp2*_(26)_, *NR1*_(24)_	15q13.3_(31)_, *MGluR5*_(4)_, *Pcdh10*_(59)_	
**Evoked delta**	*Fmr1*_(38)_	*Fmr1*_(39)_*, Mecp2*_(35)_	***Mecp2***_(26, 27)_
**Evoked theta**	*Fmr1*_(39)_*, MGluR5*_(4)_	*Fmr1, Mecp2*_(35)_	*Fmr1*_(38)_, ***Mecp2***_(26, 27)_
**Evoked alpha**		*MGluR5*_(4)_	*Ehmt1*_(9)_, ***Mecp2***_(26,27)_
**Evoked beta**	*Fmr1*_(39)_, ***Mecp2***_(27,35)_	*Fmr1, Mecp2*, *MGluR5*_(4)_	*Ehmt1*_(9)_, *Fmr1*_(38)_*, Mecp2*_(26)_
**Evoked gamma**	***Fmr1***_(37, 38, 39, 83)_, ***Mecp2***_(27, 35)_, *MGluR5*_(4)_	*Fmr1*_(83)_	15q13.3_(31)_, *Ehmt1*_(9)_, *Fmr1*_(83)_, *Mecp2*_(26)_, *NR1*_(24)_, *Pcdh10*_(59)_, VPA_(23)_
**Inter-trial gamma**	*Fmr1*_(38)_, ***Mecp2***_(27, 35)_	*Fmr1*_(39)_, *Pcdh10*_(59)_	15q13.3_(31)_, *Ehmt1*_(9)_, ***Fmr1***_(30,38, 57, 58)_, *Mecp2*_(26)_, *NR1*_(24)_, VPA _(23)_

Because of the specific interest in gamma activity in the auditory system in autism, fewer studies have investigated activity in other frequency bands. The results across these studies do not show consistent differences from WTs in resting or evoked activity [8, 20, 48, 49, 55, 64, 67–69, 101, 102, 106]. Unfortunately, there is no increase in delta and decrease in alpha which, together with the increased gamma, would have reflected the u-shaped pattern seen in people with autism and Rett syndrome [113, 144].

The most consistent phenotypes in EEG power spectra in ASD and FXS relate to resting and evoked gamma power and ITC of gamma activity. Clearly, the attention that gamma activity has raised in human studies has affected the priorities of rodent studies: while several report null results or increase for other frequency ranges, many leave them out altogether. Resting gamma power is consistently increased across rodent models, which matches the human endophenotype. Evoked gamma activity appears to be increased in *Fmr1* models, but decreased across most other rodent models, which also fits with what has been found in humans. ITC of gamma activity is also decreased in most rodent studies, which is consistent with both ASD and FXS. Gamma activity is associated with intra-regional connectivity, so the gamma frequency data reflect the internal connectivity within the auditory cortex and frontal cortex: increased at rest, decreased in response to sound (though increased in FXS), and with reduced intracortical synchrony.

#### Functional magnetic resonance imaging

Functional magnetic resonance imaging (fMRI) gives information on the activation of brain regions based on changes in blood flow to those areas, and connectivity of these areas is inferred by their co-activation. Auditory activity in people with autism as measured by fMRI has had mixed results: in some cases, activity in the auditory cortex has a sustained duration, and in others, it is only decreased compared to non-autistic people in response to speech sounds rather than simple tones [47, 81]. In *Syn2*^*−/−*^, *En2*^*−/−*^, and *Nf1*^*−/−*^ mice, the resting connectivity between the auditory cortex and the frontal cortex is decreased, and so is the connectivity within the auditory cortex and between contralateral auditory cortices, which is also observed in children with *Nf1*-associated ASD [14, 80, 127]. However, in *Chd8*^*+/−*^ mice, the resting connectivity is increased within and between auditory cortices and between the auditory cortex and subcortical structures [135]. Functional connectivity of the auditory cortex is also increased in 15q13.3 but not 22q11.2 or 1q21.1 microdeletion mice or *Iqsec2*^*−/y*^ mice [65, 108].

This is a relatively short list of studies, but fMRI shows promise as a non-invasive measure for investigating cortical connectivity at a coarser timescale but finer structural scale than EEG. More studies in humans which specifically target auditory connectivity are required to gauge whether the results in rodent models accurately recapitulate any fMRI endophenotypes, particularly with regard to different types of sound stimuli.

#### Behavioural responses

A range of tests are available to measure the behavioural output of the neural processing of auditory stimuli in rodents. One of the simplest tests is to measure the likelihood of an acoustic startle response (ASR) following auditory stimulation by measuring the degree by which the animal jumps or moves in response to the sound. The equivalent test in humans measures the degree of activity in the eyelid muscles with an electromyogram [52]. In *Fmr1*^*−/−*^ mice, there is a consistent pattern of increased ASR vigour compared to WTs at lower sound levels (70–90 dB SPL), but decreased amplitude of the ASR at higher sound levels of 100–120 dB SPL [15, 79, 92, 93]. In humans with FXS, changes in ASR have not been observed to stimuli presented at 105 dB SPL [40, 52]. According to the rodent data, this null result in humans may be due to use of an intermediate stimulus level, and future studies should investigate a wider range of sound levels to establish whether a similar bimodal effect arises in humans. In other ASD models, the results are more varied (see summary in Table 4). A rule which would explain the majority of results in studies that investigated multiple sound levels would be as follows: similar response to WTs at lower intensities, but increased startle amplitudes at higher intensities [20, 38, 41, 42, 44, 71, 84, 85, 89, 124]. This would fit with observations of increased auditory startle to loud sound stimuli in people with autism [58]. However, contradictory studies show either increased or unchanged startle amplitudes at low intensities and no difference at higher intensities [8, 61, 109, 138] or decreased startle amplitude [39, 62, 111, 143]. It is worth noting that more than half of these contradictory studies [8, 39, 62, 109, 143] were conducted in C57/BL6 mice, which is not the case for any of the studies that fit the rule. Studies which present startle stimuli at a wide range of sound levels [84, 124] are most useful for understanding this phenotype: several of the studies included only tested 110–120 dB SPL as a sound level [15, 20, 21, 38, 93, 111, 138], which does not allow detection of increased startle at sub-maximal intensities. Indeed, studies of acoustic startle in humans have also shown somewhat conflicting results in whether startle amplitude is increased or unchanged, and these studies may also benefit from using a wider range of sound levels [131]. Interestingly, some studies showed sex-related differences in ASR: in *Wnt1* and *Drd2* selective knockout mice, females have increased ASR but males are not different to WTs [71, 89], whereas in LPS-treated rats, males have increased ASR and females are unchanged from WT [38]. Sex differences are therefore another factor that should be investigated in future studies, alongside wider ranges of frequencies for startle stimuli.

**Table 4:** Summary of results from behavioural tests of auditory function in rodent models of autism. Entries are in bold font in cases where a result is represented by more than one study in a model or represents the majority of the studies in that model. Numbers in subscript brackets refer to the relevant papers from the literature search, as numbered in Supplementary Table 1. Models are listed in alphabetical order

Test	Models increased compared to WT	Models unchanged compared to WT	Models decreased compared to WT
**Acoustic startle response (ASR)** **<100 dB SPL**	***Fmr1***_(45)_, *Wnt1*_(52)_	*Cb1*_(21)_*, Chd8*_(33)_, *Cntn4*_(48)_, ***Cntnap2***_(47, 70)_, *Drd2*_(42)_, *Neph2*_(80)_, *NR1*_(22, 24)_, *Wnt1*_(52)_	*Cacnα2δ3*_(34)_
**Acoustic startle response (ASR)** **>100 dB SPL**	22q11.2_(11)_, ***Cntnap2***_(47, 70)_, *Cntn4*_(48)_, *Drd2*_(42)_, *Ehmt1*_(9)_, LPS_(19)_, ***NR1***_(22, 24)_	*Chd8*_(33)_, *Drd2*_(42)_, *MGluR5*_(4)_, *Nlgn3*_(76)_, *TS2*_(62)_, VPA_(10)_	15q13.3_(20)_, ***Fmr1***_(7, 55)_, *Neph2*_(80)_, VPA_(64)_
**Pre-pulse inhibition (PPI)**	*Cntnap2*_(77)_, ***Fmr1***_(7, 55)_, *MGluR5*_(4)_, *Ube3a*_(56)_	15q13.3_(20)_, *Drd2*_(42)_, *Cntn4*_(48)_, *Shank3b*_(63)_, *Slo1*_(79)_, *TS2*_(62)_, VPA_(10)_	22q11.2_(11)_, *Cntnap2*_(47, 70)_, *Ehmt1*_(9)_, LPS_(19)_, *Nlgn3*_(76)_, ***NR1***_(22, 24)_, VPA_(64)_
**Frequency distinction (embedded tone)**	*Cntnap2*_(77)_, *Shank3b*_(63)_, *TS2*_(62)_	*Ptchd1*_(51)_, *Ube3a*_(56)_	
**Gap detection**	*TS2*_(62)_	*Shank3b*_(63)_	*Cntnap2*_(77)_
**Complex sound distinction**		*Fmr1*_(14)_, *Mecp2*_(16)_	VPA_(13)_
**Task with background noise**			*Mecp2*_(16)_, *Ptchd1*_(51)_

More complex behavioural tasks include pre-pulse inhibition (PPI): using a quieter stimulus to cue the startle-inducing stimulus, which decreases the intensity of the startle response. The effect of the pre-pulse is consistently disrupted in people with FXS and sometimes disrupted in those with iASD [58, 78, 97, 131]. The greatest proportion of rodent models do show the expected decrease in PPI compared to WTs [20, 23, 38, 42, 44, 84, 111, 124, 138]. Interestingly, *Slo1*^*−/−*^ mice show no initial difference in PPI, but did not increase PPI with practice as observed in WTs [141]. However, several other models showed no difference in PPI [21, 39, 71, 85, 109, 110]. Still others showed an increased effect of the pre-pulse on inhibition of the startle response [8, 15, 93, 96, 140]. PPI tests therefore produce a wide range of results in different ASD models, but most commonly decreased PPI, similarly to human cohorts of iASD [58, 97]. There has been some criticism of analysis methods of PPI studies, specifically that using ANOVA statistical tests to compare PPI does not take habituation of the ASR into account, and can produce false positive results [21], and that variations in experimental setup may produce inconsistencies between research groups [125]. These may explain some of the discrepancies seen between these behavioural studies and should inform future studies for best practice on PPI paradigms. Furthermore, a study in which rats were trained in an operant conditioning paradigm with auditory cues found that the reaction time was faster in *Fmr1*^*−/−*^ than WT rats and that a longer stimulus did not improve reaction times in *Fmr1*^*−/−*^ as it did for WT rats [6]. This was interpreted by the authors as more rapid ‘temporal integration of loudness’ in the knockouts, and such a change could affect perception of the timing and loudness of the pre-pulse in a PPI paradigm, which could alter the extent of inhibition produced by the pre-pulse.

Expanding from the basic PPI framework, other paradigms can use the known rate of PPI to measure whether the pre-pulse stimulus is detectable or not. On an embedded tone task, in which a baseline background tone is presented with a tone which deviates from the baseline frequency acting as the pre-pulse, *Cntnap2*^*−/−*^ and *TS2*-neo mice showed greater attenuation of the startle response compared to WTs [109, 140]. However, *Shank3b*^*−/−*^ and *Ube3a*^*+/−*^ mice showed no difference [96, 110]. More precise tests investigate the animal’s ability to discriminate pitch by using pre-pulse tones that deviate from the baseline frequency by less than 100 Hz. In this test of pitch discrimination, *Shank3b*^*−/−*^ and *Cntnap2*^*−/−*^ mice showed improved performance compared to WTs, though *Ptchd1*^*−/−*^ and *TS2*-neo mice were not different to WTs [88, 109, 110, 140]. The pre-pulse can also take the form of a gap of silence in background white noise, and this has been used with mixed findings: *TS2*-neo mice performed better than WTs at detecting the gap, but only on the more difficult version of the test where the gap was much shorter [109]. Meanwhile, *Shank3b*^*−/−*^ mice showed no difference, and *Cntnap2*^*−/−*^ mice showed less attenuation compared to WTs [110, 140].

When VPA rats were trained to discriminate between human speech sounds, they performed worse than WTs at distinguishing consonants, but no different at distinguishing vowels [29]. However, other studies in *Fmr1*^*−/−*^ and *Mecp2*^*+/−*^ rats found no differences from WTs [30, 32]. It is difficult to make conclusions about these more complex tasks — embedded tone, gap detection, and speech sound distinction, since fewer studies have implemented them and the results do not show a clear trend. Investigations with complex sounds stand to provide different information about the auditory processing pathway in animal models of autism than those with simple sounds, so future studies are encouraged to include such stimuli.

A common trait in humans with autism is difficulty separating meaningful sounds from background noise [26, 139]. This is illustrated in several rodent studies: in *Mecp2*^*+/−*^ rats, the ability to distinguish human speech sounds is impaired compared to WTs in the first week of training (though recovers in the second and third weeks to WT levels), at a range of intensities of background noise [32]. In a behavioural task requiring mice to respond to one pure tone stimulus but not two control stimuli, *Ptchd1*^*−/−*^ mice performed equally well as WTs in the absence of background noise, but significantly worse than WTs in the presence of noise (Miho [88]). This is consistent with findings in humans with autism [26].

Behavioural studies of auditory function in rodent models of ASD highlight another difference between *Fmr1* models and other autism models. In *Fmr1*, ASR is increased at low sound levels but decreased at higher levels compared to WT, whereas in other models, ASR is unchanged at low levels but increased at higher levels. More complex behavioural tests of auditory function, such as PPI, unfortunately show inconclusive results. This variation may represent different auditory phenotypes for different aetiologies of autism, which are generally not possible to separate in studies of people with iASD in which the aetiology is unknown. The few studies that have tested performance on auditory tasks in the presence of background noise showed that this was decreased in autism model rodents.

### Invasive measures

The results from more invasive measures in the included studies provide information on the cellular functions underlying the non-invasive auditory phenotypes observed in the previous section. A presumptive model of the changes to the ascending auditory processing pathway in rodent models of autism based on these results is presented in Fig. 4. Supplementary Fig. 1 shows a complementary figure for analogous changes from human studies.

#### Histology

A major advantage of rodent studies is that histological studies can be made ex vivo in order to gain an understanding of the anatomy of auditory brain structures. Changes to the function of the auditory network can be inferred by staining for absolute number or proportion of different cell types within given regions. Additionally, immunostaining for the immediate early gene c-Fos or metabolic enzyme cytochrome oxidase (CO) in brain tissue immediately after auditory stimulation can give a proxy measure of activation of each neuron, as expression of c-Fos and activity of CO are upregulated following heightened neuronal activity [27, 149]. Changes to the volume of the auditory structures and the density of the neurons within them have been noted in rodent models of ASD: In the VCN, the overall number of neurons and number projecting to the CNIC and MGN is reduced in VPA-treated animals, and the number projecting to the MNTB is decreased in *Fmr1*^*−/−*^ rats [73, 118, 153, 154]. In *Fmr1*^*−/−*^ mice, VCN neurons fail to increase in size with age, leading to decreased size but not number of VCN neurons in adults [115, 116]. Despite these decreases, in both the VCN and the MNTB, the number of neurons shown to be active following 4-kHz or 16-kHz sound exposure was increased in VPA rats, and the typical tonotopic bands of the structure were less narrowly defined [24]. Other changes that have been observed in the MNTB include decreased overall volume in thalidomide rats, reduced number of neurons in thalidomide and VPA rats, and smaller, rounder neurons in *Fmr1*^*−/−*^ rats [53, 116, 118, 153]. Immunostaining for excitatory and inhibitory inputs to the MNTB indicated that there was increased inhibition compared to excitation in *Fmr1*^*−/−*^ mice, suggesting dampened activity of the MNTB [116]. This indicates reductions in neuronal numbers, maturity, and innervation in the MNTB, a major supplier of inhibitory input in the SOC. The number of overall neurons is also decreased in the MSO and LSO in VPA rats, and the number of inhibitory neurons was decreased in the SPON of *Fmr1*^*−/−*^ rats [118, 153]. These results are similar to studies of human brains, which have found fewer and smaller neurons in the SOC of those with ASD, especially in the MSO, where the neurons were also rounder, consistent with an immature phenotype [74, 132]. The levels of neurotransmitters glutamate, GABA, and glutamine were not changed in the SOC of *Cntnap2*^*−/−*^ rats [84].

Cell counts in VPA rats showed significantly fewer neurons in the DNLL and VNLL. The DNLL in particular also had larger, less densely packed neurons in VPA rats than WTs [75]. VPA treatment decreased the number of neurons in the CNIC, as well as decreasing its overall size and neuronal packing density, while increasing the average size of neurons [75]. Despite the loss of neurons, the IC also showed an increased number of activated neurons and a blurring of the distinctive tonotopic bands, similarly to the VCN and MNTB in VPA rats [24]. Increased activation following broadband sound exposure as measured by c-Fos staining was also seen in the IC and MGN of *Fmr1*^*−/−*^ mice [91]. Coarse analysis of the IC of *Wnt1*^*dTg*^ mice, however, showed increased volume [89]. The number and size of neurons in the MGN of *Cntnap2*^*−/−*^ mice has been shown to be reduced [140]. Despite increased sound-evoked activity shown by c-Fos staining, CO staining showed reduced activity in the IC, LL, and MGN in the absence of sound stimulation in VPA rats [86].

In the auditory cortex, the levels of GABA but not glutamate were increased in *Pchd10*^*+/−*^ mice [106]. However, in VPA rats, there are fewer inhibitory interneurons in the auditory cortex, while excitatory neurons have increased dendritic spine density [17]. The increased spine density could lead to hyperexcitability of responses to the same concentration of glutamate, but increased GABA with fewer inhibitory neurons seems unlikely, so it is unclear whether the phenotypes of these two models overlap.

Overall, anatomic studies of rodent models of ASD seem to point to fewer, smaller, and less mature neurons in subcortical auditory structures, especially those that are primarily inhibitory. This reduction in inhibition in subcortical structures may represent the origin of increased activation in higher auditory structures, leading to auditory hypersensitivity and reduced preciseness of responses.

#### Electrophysiology

Recording electrical activity from populations of neurons by placing one or more electrodes into a brain region or recording activity from single neurons via whole cell patch-clamp provides readout of neuronal activity with high temporal resolution. Electrophysiological recordings can be made either in vivo in response to sounds or in acute tissue slices in response to electrical stimulation. A summary of the results of electrophysiological studies, as well as other functional studies, on the overall changes to the auditory pathway can be seen in Fig. 4. In the SOC, few differences have been found in electrical responses in the MNTB in *Tsc1*^*+/−*^, *Nf1*^*+/−*^, and *H-ras* mutant mice [145]. The rate of spontaneous activity and evoked activity and the frequency of miniature excitatory post-synaptic potentials (mEPSPs) were unchanged, though in the *Nf1*^*+/−*^ mice the latency to a response at the major relay site of the calyx of Held was decreased [145]. In the LSO of *Fmr1*^*−/−*^ mice, the evoked firing rate and mEPSP frequency are increased, and the frequency tuning of neurons in response to pure tones was broader, indicating hyperactivity and reduced specificity of neuronal responses [45]. In the SPON of *Chrna7*^*−/−*^ mice, the rate of spontaneous activity is not different from WTs, but the response latency following sound onset was increased, and the resulting activity lasted longer. The length required for a silent gap in a train of sound to be detected in recordings of SPON activity was increased in the *Chrna7*^*−/−*^ mice [37]. This evidence points to little change in the activity of the MNTB in ASD, but hyperactivity of the LSO and delayed responses in the SPON.

In the IC, frequency tuning was broader in *Fmr1*^*−/−*^ mice, especially for lower frequencies, but unchanged in *Chrna7*^*−/−*^ or *Cacnα2δ3*^*−/−*^ mice [11, 37, 91]. The spontaneous firing rate was increased in *Fmr1*^*−/−*^ and *Cacnα2δ3*^*−/−*^ mice, and evoked firing rates were increased in *Fmr1*^*−/−*^ mice [11, 91]. The latency to respond was increased in *Chrna7*^*−/−*^ mice, which carried through to the latency to respond in the VNLL [37]. Responses in the IC of *Cacnα2δ3*^*−/−*^ mice followed slower sound presentation rates (10–30 per second), but were poorly coupled to presentation of auditory stimuli at a faster rate of 100 per second [11]. In the auditory thalamus of *Ptchd1*^*−/−*^ mice, the spontaneous firing rate increased in the TRN but not the MGN. The evoked firing rate is increased in both the TRN and MGN, and the presence of background noise further increases the firing rate in the MGN and impairs the ability of MGN neurons to follow rapid sounds [88]. This fits with the behavioural phenotype of poorer performance on auditory tasks in the presence of background noise [32, 88].

Electrophysiological recordings from the auditory cortex are more prevalent than from lower auditory structures. Results from these studies can vary, for example the spontaneous firing rate is decreased in *Shank3*^*+/−*^ and *Cntnap2*^*−/−*^ rat auditory cortex, but increased in *Fmr1*^*−/−*^ rat auditory cortex, and unchanged in ex vivo slice recordings from *Fmr1*^*−/−*^ mice [33, 123, 133, 146]. Another measure of hyperexcitability is an increased ratio of mEPSPs to miniature inhibitory post-synaptic potentials (mIPSPs). A higher frequency of mEPSPs than mIPSPs compared to WTs has been observed in A1 of VPA mice, *Ehmt1*^*+/−*^ mice and *Pten* conditional knockout mice, indicating underlying hypersensitivity of the AC [87, 90, 150]. The evoked firing rate is decreased in *Shank3*^*+/−*^ rats, VPA rats, and *Fmr1*^*−/−*^ rats, but increased in *Mecp2*^*+/−*^ and *Cntnap2*^*-/−*^ rats, *Fmr1*^*−/−*^ mice (particularly in layers 2/3), and *Mecp2* overexpressing mice [28, 30, 32, 33, 117, 123, 146, 152]. Interestingly, in vivo studies in VPA rats show no change in evoked firing rate to a simple 5-kHz tone, but decreased firing rates to human speech sounds, which fits with the patterns of evoked activity measured by fMRI in humans with autism [7, 28, 29, 47]. Mice with a conditional knockout of *Pten* after the auditory critical window displayed a decreased firing rate compared to WTs at low levels of stimulation, but increased at higher levels [150]. One study in *Fmr1*^*−/−*^ mice showed that the divergence from WTs in evoked activity changes with age, likely related to effects of the mutation on the timing of reversal of GABA receptor polarisation [133]. Differences in the level of activity in the auditory cortex in various models may therefore be affected by different excitability of lower auditory structures in each model, and by the stimulus intensity in ex vivo and complexity of the sound in in vivo experiments. Future studies of spontaneous and evoked activity should ideally compare these over a wide range of stimulus intensities and ages to better characterise AC development in their models.

The frequency tuning of A1 neurons in VPA rats, *Fmr1*^*−/−*^ mice, and *Mecp2* overexpressing mice, and the AAF of VPA rats, is broader, indicating reduced specificity of responses and hyperexcitability [4, 28, 117, 152]. Neurons in the auditory cortex of *Fmr1*^*−/−*^ mice also show less specificity for a preferred speed of frequency sweep stimulus [117]. The degree of cortical tonotopy, a measure of functional frequency response organisation, has been found to be reduced in VPA rat AAF and reduced or unchanged in VPA rat A1 [4, 28]. Beyond the amplitude of the response and the specificity of A1 neurons, the speed of transmission to the auditory cortex was also measured in several studies. The latency of responses is increased in A1 of *Mecp2*^*+/−*^ rats and *Mecp2* overexpressing mice, and in the AAF of VPA rats, though it is decreased in the A1 of VPA rats and not affected at all in *Shank3*^*−/−*^ rats [4, 28, 32, 33, 152]. Speed of transmission likely also leads to the inability of auditory cortex neurons to follow along with rapidly presented sounds in *Mecp2*^*+/−*^, *Cntnap2*^*−/−*^, and VPA rats [17, 32, 123]. This characteristic resembles the activity of A1 in younger WT rats, indicating this may represent an ‘immaturity’ of the auditory cortex in these animals [123]. VPA-treated rats also more poorly distinguish between different rates of sound stimulus presentation in behavioural tasks, which is likely linked to this electrophysiological finding [17]. Based on the results from the IC of *Cacnα2δ3*^*−/−*^ mice, this auditory cortex phenotype may arise subcortically [11].

Overall, while there are some conflicts between results in electrophysiological studies, there is convergent evidence for broader frequency tuning of neurons in several structures along the auditory processing pathway and increases in spontaneous and evoked firing rates of neurons in lower auditory structures, corresponding to hyper-excitation within these structures. Activity in the auditory cortex may change with age, which may explain some variability in results [56]. There is evidence that complex sounds are more likely to elicit differences from WTs than simple sounds and that ASD models have degraded temporal precision of responses, especially in the presence of background noise [7, 28, 32, 88]. These results fit well with equivalent measures of responses to simple and complex sounds, as well as sounds with background noise, in people with autism [26, 47, 139].

## Conclusions

The recent expansion in the variety of rodent models of autism used in auditory studies provides a significantly richer framework for comparing endophenotypes among mice, rats, and humans and has provided more insight into how the auditory processing circuit develops and functions differently in ASD to produce these phenotypes. However, this large array of models presents a pool of results that are more complex and diverse than is the case in single-gene disorders. In the same way that human studies with a range of aetiologies present varying results in many auditory tests, comparing across an array of different rodent models here has frequently presented conflicting results. There are, however, some convergent auditory endophenotypes across the studies represented in this review, which match findings in humans. These include the increased latency of peaks in the ERP of ASD models (though the decreased amplitude of the N1 peak seen in human ASD was not well recapitulated) and increased amplitude of the N1 peak in *Fmr1* models, which matches the divergence in FXS from the typical human autism endophenotype. Likewise, the power of gamma activity in the auditory and frontal cortices is affected differently in FXS and iASD. Increased resting gamma power in both *Fmr1* and other autism models and increased evoked gamma power in *Fmr1* models but decreased across other models match well with recordings from human studies. Decreased inter-trial coherence of gamma activity was also consistently reduced, which matches well with human endophenotypes. Based on the rodent studies summarised in this review, sound intensity-dependent changes to the ASR show promise as an endophenotype which may warrant more in-depth investigation in humans with autism and FXS, as this is evidently another auditory endophenotype which separates the two groups. More complex behavioural tasks show merit but are thus far inconclusive.

In the more invasive studies, the most consistent result from histology in rodent ASD models is a lack of maturation and sometimes number of neurons in various auditory structures, with inhibitory interneurons being the most affected. A common theme across electrophysiology studies is broader frequency tuning of neurons: responding to a wider range of frequencies and therefore showing less specificity to any given frequency of sound. Both findings point to hypersensitivity and dysregulation in the auditory system, which ties to the increased intensity of the ASR and decreased inter-trial coherence of gamma activity in the auditory cortex.

Interestingly, several studies spanning histology, electroencephalography, and electrophysiology noted phenotypes in rodent models of autism that were ‘immature’ or resembled that of a younger WT animal. Other studies found phenotypic differences were not present in older animals, suggesting that differences between the WTs and autism model animals may represent different developmental trajectories rather than finite differences. There is even more reason, therefore, to recommend testing animals at a wide range of ages.

To broadly summarise the effects of changes in the auditory system in ASD, there is increased excitation in subcortical structures and less precise tuning of neurons to specific frequencies. The connectivity within the auditory cortex is increased, but connectivity between the auditory cortex and other brain regions is decreased. The effect of these changes is increased perception of and ability to distinguish simple sounds, but reduced perception of complex sounds and sounds in the presence of background noise. Future studies should note the endophenotypes that have been shown to be the most successful in comparing auditory processing between humans and rodent models of ASD. These include the amplitude and latency of the N1 peak, power, and coherence of gamma activity in the auditory cortex at rest and evoked by sound, acoustic startle at high sound levels, and ability to distinguish sounds in the presence of background noise.

### Limitations and future directions

One reason for the observed conflicting results may be due to differences in experimental conditions. Studies which used the same paradigms across different rodent models can claim more conclusively that differences in the results are truly due to differences in phenotypes between models [13, 28, 32, 145]. One way to improve replicability in this field may be to standardise experiments in order to make them more comparable between laboratories and different ASD models, in a manner similar to the International Brain Laboratory [2]. We have presented suggestions for improving auditory phenotyping, such as comparing a wider range of sound levels across specific developmental and ageing stages, and measuring cortical ERPs in awake, rather than anaesthetised animals. Other recommendations for standardisation have been made in two recent reviews regarding translational potential of rodent models of autism, including repeating our recommendation of comparisons across specific ranges of ages, particularly younger ones [125, 128]. While results from human studies should be used to gauge whether a rodent model accurately reflects relevant endophenotypes [83], results from these rodent models can also reveal new avenues to inform future directions of human studies. For example, we have highlighted that differences in the auditory startle are sound level-dependent in both *Fmr1* and iASD rodent models, and testing a range of sound intensities in human studies may provide a better understanding of differences in the ASR of the relevant human populations.

While *Fmr1* rodent models are well established and present attractive model organisms due to their relative genetic simplicity, researchers should use caution in using these models as a proxy for iASD in auditory studies. While the co-occurrence of FXS and autism is high, phenotypes such as increased evoked gamma power in the auditory cortex are very much FXS-specific, and well conserved in rodent models of FXS. For this reason, we have tried to compare across non-FXS models in this review to represent modelling of iASD auditory endophenotypes. Further, a concerning number of studies used mouse models on a C57BL/6 genetic background, which has a known phenotype of hearing loss after 3 months of age [95]. We have seen in this review that mouse autism models on this background over 3 months old often have auditory phenotypes that diverge from the majority of animals from other strains. The recommendation to conduct experiments in younger mice is therefore twofold if they are of this genetic background, and we advise future studies move towards using other strains.

This review has brought together the existing research into the central auditory function of a wide range of rodent models of autism. While some measures of auditory function show inconsistent results across studies, others have emerged as strong endophenotypes across models which also recapitulate auditory endophenotypes from humans with autism. Other studies have provided insight into the underlying cellular changes driving these endophenotypes. We hope that this review will be informative for further studies with regard to existing data and best practices for future studies, to ultimately further understanding of the development of the auditory system in human autism.

## Supplementary Information

**Additional file 1: Supplementary Table 1.** Primary research articles which passed the criteria for inclusion in the systematic review. Studies conducted using mouse models on the C57/Black6 background at an age over 3 months are indicated by an asterisk after the author name. **Supplementary Table 2.** Rodent models with auditory phenotypes described in this review. Gene names are formatted as in the cited papers, with alternate names for equivalent human genes used in SFARI gene in brackets where relevant. **Supplementary Figure 1.** The ascending auditory processing pathway with presumed changes in autism based on results from human studies. Excitatory (red) and inhibitory (blue) connections between structures along the pathway are illustrated along with the changes to these connections and the activity within areas in human autism studies. Connections are primarily ipsilateral unless otherwise stated. Responses in the superior olivary complex occur later, likely due to slower transmission from the cochlear nuclei. Responses in the inferior colliculus also occur later, but it is unclear whether it is input from the cochlear nuclei or the superior olivary complex, or both, which is delayed. Signals from the auditory thalamus take longer to reach the auditory cortex in human autism papers. There is decreased activity and increased latency (N1), and reduced gamma in the auditory cortex in human autism studies.

## Data Availability

Not applicable.

## Abbreviations

ASD: Autism spectrum disorders
EEG: Electroencephalography
MEG: Magnetoencephalography
fMRI: Functional magnetic resonance imaging
ABRs: Auditory brainstem responses
ERPs: Cortical event-related potentials
FXS: Fragile X syndrome
iASD: Idiopathic autism spectrum disorder
DCN: Dorsal cochlear nucleus
VCN: Ventral cochlear nucleus
AVCN: Anterior VCN
PVCN: Posterior VCN
SOC: Superior olivary complex
MSO: Medial superior olive
LSO: Lateral superior olive
MNTB: Medial nucleus of the trapezoid body
LNTB: Lateral nucleus of the trapezoid
SPON: Superior paraolivary nucleus
LL: Lateral lemniscus
DNLL: Dorsal nucleus lateral lemniscus
VNLL: Ventral nucleus lateral lemniscus
IC: Inferior colliculus
CNIC: Central nucleus of the inferior colliculus
MGN: Medial geniculate nucleus
TRN: Thalamic reticular nucleus
A1: Primary auditory cortex
A2: Secondary auditory cortex
AAF: Anterior auditory field
PFC: Prefrontal cortex
VPA: Valproic acid
LPS: Lipopolysaccharide
ITC: Inter-trial coherence
ASR: Acoustic startle response
PPI: Pre-pulse inhibition
mEPSPs: Miniature excitatory post-synaptic potentials
mIPSPs: Miniature inhibitory post-synaptic potentials
