# Supplementary material for: Auditory processing in rodent models of autism: a systematic review
Source: J Neurodev Disord. 2022 Aug 30;14:48. doi: 10.1186/s11689-022-09458-6 (PMC9429780; doi:10.1186/s11689-022-09458-6)

Supplementary table 1: Primary research articles which passed the criteria for inclusion in the systematic review. Studies conducted using mouse models on the C57/Black6 background at an age over 3 months are indicated by an asterisk after the author name.

| **#** | **Authors** | **Title** | **Year** |
| --- | --- | --- | --- |
| 1 | Anomal et al. | Impaired Processing in the Primary Auditory Cortex of an Animal Model of Autism | 2015 |
| 2 | Auerbach et al. | Auditory hypersensitivity and processing deficits in a rat model of fragile X syndrome | 2021 |
| 3 | Banerjee et al. | Abnormal emotional learning in a rat model of autism exposed to valproic acid in utero | 2014 |
| 4 | Barnes et al.  * | Disruption of mGluR5 in parvalbumin-positive interneurons induces core features of neurodevelopmental disorders | 2015 |
| 5 | Bracic et al.  * | Impaired Subcortical Processing of Amplitude-Modulated Tones in Mice Deficient for Cacna2d3, a Risk Gene for Autism Spectrum Disorders in Humans | 2022 |
| 6 | Chelini et al.  * | Aberrant Somatosensory Processing and Connectivity in Mice Lacking Engrailed-2 | 2019 |
| 7 | Chen and Toth | Fragile X mice develop sensory hyperreactivity to auditory stimuli | 2001 |
| 8 | Cheng et al. | Degraded cortical temporal processing in the valproic acid-induced rat model of autism | 2022 |
| 9 | Davis et al. | Impairments in sensory-motor gating and information processing in a mouse model of Ehmt1 haploinsufficiency | 2020 |
| 10 | Degroote et al. | Improved assessment of sensorimotor gating in animal models relevant to ASD: A data modelling approach to quantify PrePulse Inhibition of the acoustic startle reflex | 2017 |
| 11 | Didriksen et al. | Persistent gating deficit and increased sensitivity to NMDA receptor antagonism after puberty in a new mouse model of the human 22q11.2 microdeletion syndrome: a study in male mice | 2017 |
| 12 | Dubiel and Kulesza | Prenatal Valproic Acid exposure disrupts tonotopic c-FOS expression in the rat brainstem | 2015 |
| 13 | Engineer et al. | Degraded Auditory Processing in a Rat Model of Autism Limits the Speech Representation in Non-Primary Auditory Cortex | 2014 |
| 14 | Engineer et al. | Degraded speech sound processing in a rat model of fragile X syndrome | 2014 |
| 15 | Engineer et al. | Speech sound discrimination training improves auditory cortex responses in a rat model of autism | 2014 |
| 16 | Engineer et al. | Degraded neural and behavioral processing of speech sounds in a rat model of Rett syndrome | 2015 |
| 17 | Engineer et al. | Shank3-deficient rats exhibit degraded cortical responses to sound | 2018 |
| 18 | Felix et al.  * | Nicotinic acetylcholine receptor subunit alpha(7)-knockout mice exhibit degraded auditory temporal processing | 2019 |
| 19 | Foley et al. | Sexually dimorphic effects of prenatal exposure to lipopolysaccharide, and prenatal and postnatal exposure to propionic acid, on acoustic startle response and prepulse inhibition in adolescent rats: relevance to autism spectrum disorders | 2015 |
| 20 | Forsingdal et al.  * | 15q13.3 homozygous knockout mouse model display epilepsy-, autism- and schizophrenia-related phenotypes | 2016 |
| 21 | Fyke et al. | Communication and social interaction in the cannabinoid-type 1 receptor null mouse: Implications for autism spectrum disorder | 2021 |
| 22 | Gandal et al. | Mice with reduced NMDA receptor expression: more consistent with autism than schizophrenia? | 2012 |
| 23 | Gandal et al. | Validating gamma Oscillations and Delayed Auditory Responses as Translational Biomarkers of Autism | 2010 |
| 24 | Gandal et al. | GABA(B)-mediated rescue of altered excitatory-inhibitory balance, gamma synchrony and behavioral deficits following constitutive NMDAR-hypofunction | 2012 |
| 25 | Garcia-Pino et al. | Enhanced Excitatory Connectivity and Disturbed Sound Processing in the Auditory Brainstem of Fragile X Mice | 2017 |
| 26 | Goffin et al. | Rett syndrome mutation MeCP2 T158A disrupts DNA binding, protein stability and ERP responses | 2011 |
| 27 | Goffin et al.  * | Cellular origins of auditory event-related potential deficits in Rett syndrome | 2014 |
| 28 | Hacohen-Kleiman et al. | Atypical Auditory Brainstem Response and Protein Expression Aberrations Related to ASD and Hearing Loss in the Adnp Haploinsufficient Mouse Brain | 2019 |
| 29 | Ida-Eto et al. | Mechanism of auditory hypersensitivity in human autism using autism model rats | 2017 |
| 30 | Jonak et al. | Multielectrode array analysis of EEG biomarkers in a mouse model of Fragile X Syndrome | 2020 |
| 31 | Kogan et al.  * | Mouse Model of Chromosome 15q13.3 Microdeletion Syndrome Demonstrates Features Related to Autism Spectrum Disorder | 2015 |
| 32 | Kulinich et al. | Beneficial effects of sound exposure on auditory cortex development in a mouse model of Fragile X Syndrome | 2020 |
| 33 | Kweon et al. | Excitatory neuronal CHD8 in the regulation of neocortical development and sensory-motor behaviors | 2021 |
| 34 | Landmann et al.  * | Behavioral phenotyping of calcium channel (CACN) subunit alpha 2 delta 3 knockout mice: Consequences of sensory cross-modal activation | 2019 |
| 35 | Liao et al.  * | MeCP2+/- mouse model of RTT reproduces auditory phenotypes associated with Rett syndrome and replicate select EEG endophenotypes of autism spectrum disorder | 2012 |
| 36 | Lichtman et al.  * | Structural and functional brain-wide alterations in A350V Iqsec2 mutant mice displaying autistic-like behavior | 2021 |
| 37 | Lovelace et al. | Translation-relevant EEG phenotypes in a mouse model of Fragile X Syndrome | 2018 |
| 38 | Lovelace et al. | Minocycline Treatment Reverses Sound Evoked EEG Abnormalities in a Mouse Model of Fragile X Syndrome | 2020 |
| 39 | Lovelace et al. | Deletion of Fmr1 from Forebrain Excitatory Neurons Triggers Abnormal Cellular, EEG, and Behavioral Phenotypes in the Auditory Cortex of a Mouse Model of Fragile X Syndrome | 2020 |
| 40 | Lovelace et al. | Matrix metalloproteinase-9 deletion rescues auditory evoked potential habituation deficit in a mouse model of Fragile X Syndrome | 2016 |
| 41 | Lukose et al. | Malformation of the superior olivary complex in an animal model of autism | 2011 |
| 42 | Lyon et al.  * | Sex-Specific Role for Dopamine Receptor D2 in Dorsal Raphe Serotonergic Neuron Modulation of Defensive Acoustic Startle and Dominance Behavior | 2020 |
| 43 | Mansour et al. | Auditory Midbrain Hypoplasia and Dysmorphology after Prenatal Valproic Acid Exposure | 2019 |
| 44 | Mansour et al. | Abnormal morphology and subcortical projections to the medial geniculate in an animal model of autism | 2021 |
| 45 | Michalon et al. | Chronic Pharmacological mGlu5 Inhibition Corrects Fragile X in Adult Mice | 2012 |
| 46 | Michetti et al. | The Knockout of Synapsin II in Mice Impairs Social Behavior and Functional Connectivity Generating an ASD-like Phenotype | 2017 |
| 47 | Möhrle et al. | GABA(B) Receptor Agonist R-Baclofen Reverses Altered Auditory Reactivity and Filtering in the Cntnap2 Knock-Out Rat | 2021 |
| 48 | Molenhuis et al. | Limited impact of Cntn4 mutation on autism-related traits in developing and adult C57BL/6J mice | 2016 |
| 49 | Morel et al. | Head-to-Head Study of Developmental Neurotoxicity and Resultant Phenotype in Rats: α-Hexabromocyclododecane versus Valproic Acid, a Recognized Model of Reference for Autism Spectrum Disorders | 2022 |
| 50 | Nagode et al. | Abnormal Development of the Earliest Cortical Circuits in a Mouse Model of Autism Spectrum Disorder | 2017 |
| 51 | Nakajima et al.  * | Combinatorial Targeting of Distributed Forebrain Networks Reverses Noise Hypersensitivity in a Model of Autism Spectrum Disorder | 2019 |
| 52 | Nakajima et al. | Dilation of the inferior colliculus and hypersensitivity to sound in Wnt1-cre and Wnt1-GAL4 double-transgenic mice | 2014 |
| 53 | Negwer et al. | EHMT1 regulates Parvalbumin-positive interneuron development and GABAergic input in sensory cortical areas | 2020 |
| 54 | Nguyen et al. | Abnormal development of auditory responses in the inferior colliculus of a mouse model of Fragile X Syndrome | 2020 |
| 55 | Olmos-Serrano et al. | The GABA(A) Receptor Agonist THIP Ameliorates Specific Behavioral Deficits in the Mouse Model of Fragile X Syndrome | 2011 |
| 56 | Perrino et al. | Communication-related assessments in an Angelman syndrome mouse model | 2021 |
| 57 | Pirbhoy et al. | Increased 2-arachidonoyl-sn-glycerol levels normalize cortical responses to sound and improve behaviors in Fmr1 KO mice | 2021 |
| 58 | Pirbhoy et al. | Acute pharmacological inhibition of matrix metalloproteinase-9 activity during development restores perineuronal net formation and normalizes auditory processing in Fmr1 KO mice | 2020 |
| 59 | Port et al.  * | Protocadherin 10 alters gamma oscillations, amino acid levels, and their coupling; baclofen partially restores these oscillatory deficits | 2017 |
| 60 | Reinhard et al. | Reduced perineuronal net expression in Fmr1 KO mice auditory cortex and amygdala is linked to impaired fear-associated memory | 2019 |
| 61 | Reinwald et al. | Separable neural mechanisms for the pleiotropic association of copy number variants with neuropsychiatric traits | 2020 |
| 62 | Rendall et al.  * | Auditory processing enhancements in the TS2-neo mouse model of Timothy Syndrome, a rare genetic disorder associated with autism spectrum disorders | 2017 |
| 63 | Rendall et al.  * | Shank3B mutant mice display pitch discrimination enhancements and learning deficits | 2019 |
| 64 | Reynolds et al. | Sensory and Motor Characterization in the Postnatal Valproate Rat Model of Autism | 2012 |
| 65 | Rotschafer and Razak | Altered auditory processing in a mouse model of fragile X syndrome | 2013 |
| 66 | Rotschafer and Cramer | Developmental Emergence of Phenotypes in the Auditory Brainstem Nuclei of Fmr1 Knockout Mice | 2017 |
| 67 | Rotschafer et al. | Deletion of Fmr1 Alters Function and Synaptic Inputs in the Auditory Brainstem | 2015 |
| 68 | Ruby et al. | Abnormal neuronal morphology and neurochemistry in the auditory brainstem of FMR1 knockout rats | 2015 |
| 69 | Saunders et al. | Knockout of NMDA Receptors in Parvalbumin Interneurons Recreates Autism-Like Phenotypes | 2013 |
| 70 | Scott et al. | Altered Auditory Processing, Filtering, and Reactivity n the Cntnap2 Knock-Out Rat Model for Neurodevelopmental Disorders | 2018 |
| 71 | Scott et al. | Hyperexcitable and Immature-Like Neuronal Activity in the Auditory Cortex of Adult Rats Lacking the Language-Linked CNTNAP2 Gene | 2022 |
| 72 | Shofty et al. | Autism-associated Nf1 deficiency disrupts corticocortical and corticostriatal functional connectivity in human and mouse | 2019 |
| 73 | Sinclair et al. | GABA-B Agonist Baclofen Normalizes Auditory-Evoked Neural Oscillations and Behavioral Deficits in the Fmr1 Knockout Mouse Model of Fragile X Syndrome | 2017 |
| 74 | Song et al. | Dysregulation of GABAA Receptor-Mediated Neurotransmission during the Auditory Cortex Critical Period in the Fragile X Syndrome Mouse Model | 2021 |
| 75 | Suetterlin et al. | Altered Neocortical Gene Expression, Brain Overgrowth and Functional Over-Connectivity in Chd8 Haploinsufficient Mice | 2018 |
| 76 | Thomas et al. | Sleep/Wake Physiology and Quantitative Electroencephalogram Analysis of the Neuroligin-3 Knockout Rat Model of Autism Spectrum Disorder | 2017 |
| 77 | Truong et al.  * | Auditory Processing and Morphological Anomalies in Medial Geniculate Nucleus of Cntnap2 Mutant Mice | 2015 |
| 78 | Tsugiyama et al. | Altered neuronal activity in the auditory brainstem following sound stimulation in thalidomide-induced autism model rats | 2020 |
| 79 | Typlt et al. | Mice with Deficient BK Channel Function Show Impaired Prepulse Inhibition and Spatial Learning, but Normal Working and Spatial Reference Memory | 2013 |
| 80 | Völker et al.  * | Neph2/Kirrel3 regulates sensory input, motor coordination, and home-cage activity in rodents | 2018 |
| 81 | Wang et al. | In vivo synaptic transmission and morphology in mouse models of Tuberous sclerosis, Fragile X syndrome, Neurofibromatosis type 1, and Costello syndrome | 2020 |
| 82 | Wen et al. | Genetic Reduction of Matrix Metalloproteinase-9 Promotes Formation of Perineuronal Nets Around Parvalbumin-Expressing Interneurons and Normalizes Auditory Cortex Responses in Developing Fmr1 Knock-Out Mice | 2018 |
| 83 | Wen et al. | Developmental Changes in EEG Phenotypes in a Mouse Model of Fragile X Syndrome | 2019 |
| 84 | Xiong et al. | PTEN Regulation of Local and Long-Range Connections in Mouse Auditory Cortex | 2012 |
| 85 | Yang et al. | 16p11.2 Deletion Syndrome Mice Display Sensory and Ultrasonic Vocalization Deficits During Social Interactions | 2015 |
| 86 | Zhou et al. | Atypical Response Properties of the Auditory Cortex of Awake MECP2-Overexpressing Mice | 2019 |
| 87 | Zimmerman et al. | Repeated Prenatal Exposure to Valproic Acid Results in Auditory Brainstem Hypoplasia and Reduced Calcium Binding Protein Immunolabeling | 2018 |
| 88 | Zimmerman et al. | In utero exposure to valproic acid disrupts ascending projections to the central nucleus of the inferior colliculus from the auditory brainstem | 2020 |

Supplementary table 2: Rodent models with auditory phenotypes described in this review. Gene names are formatted as in the cited papers, with alternate names for equivalent human genes used in SFARI gene in brackets where relevant.

| Genetic rodent models of ASD: single genes | | | |
| --- | --- | --- | --- |
| Gene | Syndrome | Role of protein | Relevant papers |
| *Adnp* | Helsmoortel-Van der Aa syndrome | Chromatin remodelling transcription factor | (Hacohen-Kleiman et al., 2019) |
| *Cacnα2δ3* | n/a | Voltage dependent Ca^2+^ channel subunit | (Bracic et al., 2022; Landmann et al., 2019) |
| *Cb1 (CNR1)* | n/a | Cannabinoid receptor | (Fyke et al., 2021) |
| *Chd8* | n/a | Chromatin remodelling | (Kweon et al., 2021; Suetterlin et al., 2018) |
| *Chrna7* | n/a | Acetylcholine receptor subunit | (Felix et al., 2019) |
| *Cntn4* | n/a | Cell adhesion, axon connections | (Molenhuis et al., 2016) |
| *Cntnap2* | Pitt Hopkins-like syndrome 1 | K^+^ channel placement, cell adhesion | (Möhrle et al., 2021; Scott et al., 2018, 2022; Truong et al., 2015) |
| *Drd2* | Tourette syndrome | Dopamine receptor | (Lyon et al., 2020) |
| *Ehmt1* | Kleefstra syndrome | Transcription repression | (Davis et al., 2020; Negwer et al., 2020) |
| *En2* | n/a | Transcription factor | (Chelini et al., 2019) |
| *Fmr1* | Fragile X syndrome | Regulation of translation, mRNA transport | (Auerbach et al., 2021; Chen and Toth, 2001; Engineer et al., 2014a; Garcia-Pino et al., 2017; Jonak et al., 2020; Kulinich et al., 2020; Lovelace et al., 2016, 2018, 2020b, 2020a; Michalon et al., 2012; Nguyen et al., 2020; Olmos-Serrano et al., 2011; Pirbhoy et al., 2020, 2021; Reinhard et al., 2019; Rotschafer and Cramer, 2017; Rotschafer and Razak, 2013; Rotschafer et al., 2015; Ruby et al., 2015; Sinclair et al., 2017; Song et al., 2021; Wang et al., 2015; Wen et al., 2018, 2019) |
| *H-ras (HRAS)* | Costello syndrome | GTP binding | (Wang et al., 2015) |
| *Iqsec2* | n/a | Post-synaptic trafficking of glutamatergic synapses | (Lichtman et al., 2021) |
| *Mecp2* | Rett syndrome | Synaptic maintenance, DNA binding | (Engineer et al., 2015; Goffin et al., 2012, 2014; Liao et al., 2012; Zhou et al., 2019) |
| *MGluR5 (GRM5)* | n/a | Glutamate receptor subunit | (Barnes et al., 2015) |
| *Neph2 (KIRREL3)* | n/a | Cell adhesion, synapse maturation | (Völker et al., 2018) |
| *Nf1* | n/a | Suppression of cell proliferation | (Shofty et al., 2019; Wang et al., 2015) |
| *Nlgn3* | n/a | Transmembrane scaffolding | (Thomas et al., 2017) |
| *NR1* | n/a | NMDA receptor subunit 1 | (Gandal et al., 2012a, 2012b; Saunders et al., 2013) |
| *Pcdh10* | n/a | Cell adhesion, cell migration | (Port et al., 2017) |
| *Ptchd1* | n/a | TRN development | (Nakajima et al., 2019) |
| *Pten* | Cowden syndrome | Suppression of cell growth | (Xiong et al., 2012) |
| *Shank3b* | Phelan-McDermid syndrome | Post-synaptic maintenance and maturation | (Engineer et al., 2018; Rendall et al., 2018) |
| *Slo1 (KCNMA1)* | n/a | K^+^ channel subunit | (Typlt et al., 2013) |
| *Syn2* | n/a | Presynaptic neurotransmitter release | (Michetti et al., 2017) |
| *TS2 (CACNA1C)* | Timothy Syndrome | Voltage dependent Ca^2+^ channel subunit | (Rendall et al., 2017) |
| *Tsc1* | n/a | Suppression of cell growth | (Wang et al., 2015) |
| *Ube3a* | Angelman syndrome | Protein degradation | (Perrino et al., 2020) |
| *Wnt1* | n/a | Central nervous system development | (Nakajima et al., 2014) |
| Genetic rodent models of ASD: microdeletions | | | |
| Deletion | Genes affected | | Relevant papers |
| 1q21.1 | *Chd1* | | (Reinwald et al., 2020) |
| 15q13.3 | *Chrna7, Fan1, Otud7a, Trpm1* | | (Forsingdal et al., 2016; Kogan et al., 2015; Reinwald et al., 2020) |
| 16p11.2 | *Bckdk, Coro1a, Kctd13, Mapk3, Setd1a, Sez6l2, Srcap, Taok2* | | (Yang et al., 2015) |
| 22q11.2 | *Tbx1* | | (Didriksen et al., 2017; Reinwald et al., 2020) |
| Environmental rodent models of ASD | | | |
| Chemical | Delivery | Origin | Relevant papers |
| Lipopolysaccharide | Prenatal and postnatal | Maternal gut inflammation | (Foley et al., 2015) |
| Thalidomide | Prenatal | Maternal prescription | (Ida-Eto et al., 2017; Tsugiyama et al., 2020) |
| Valproic acid | Prenatal and postnatal | Maternal prescription | (Anomal et al., 2015; Banerjee et al., 2014; Cheng et al., 2022; Degroote et al., 2017; Dubiel and Kulesza, 2015; Engineer et al., 2014c, 2014b; Gandal et al., 2010; Lukose et al., 2011; Mansour et al., 2020, 2019; Morel et al., 2022; Nagode et al., 2017; Reynolds et al., 2012; Wang et al., 2015; Zimmerman et al., 2018, 2020) |

Supplementary Figure 1: The ascending auditory processing pathway with presumed changes in autism based on results from human studies. Excitatory (red) and inhibitory (blue) connections between structures along the pathway are illustrated along with the changes to these connections and the activity within areas in human autism studies. Connections are primarily ipsilateral unless otherwise stated. Responses in the superior olivary complex occur later, likely due to slower transmission from the cochlear nuclei. Responses in the inferior colliculus also occur later, but it is unclear whether it is input from the cochlear nuclei or the superior olivary complex, or both, which is delayed. Signals from the auditory thalamus take longer to reach the auditory cortex in human autism papers. There is decreased activity and increased latency (N1), and reduced gamma in the auditory cortex in human autism studies.


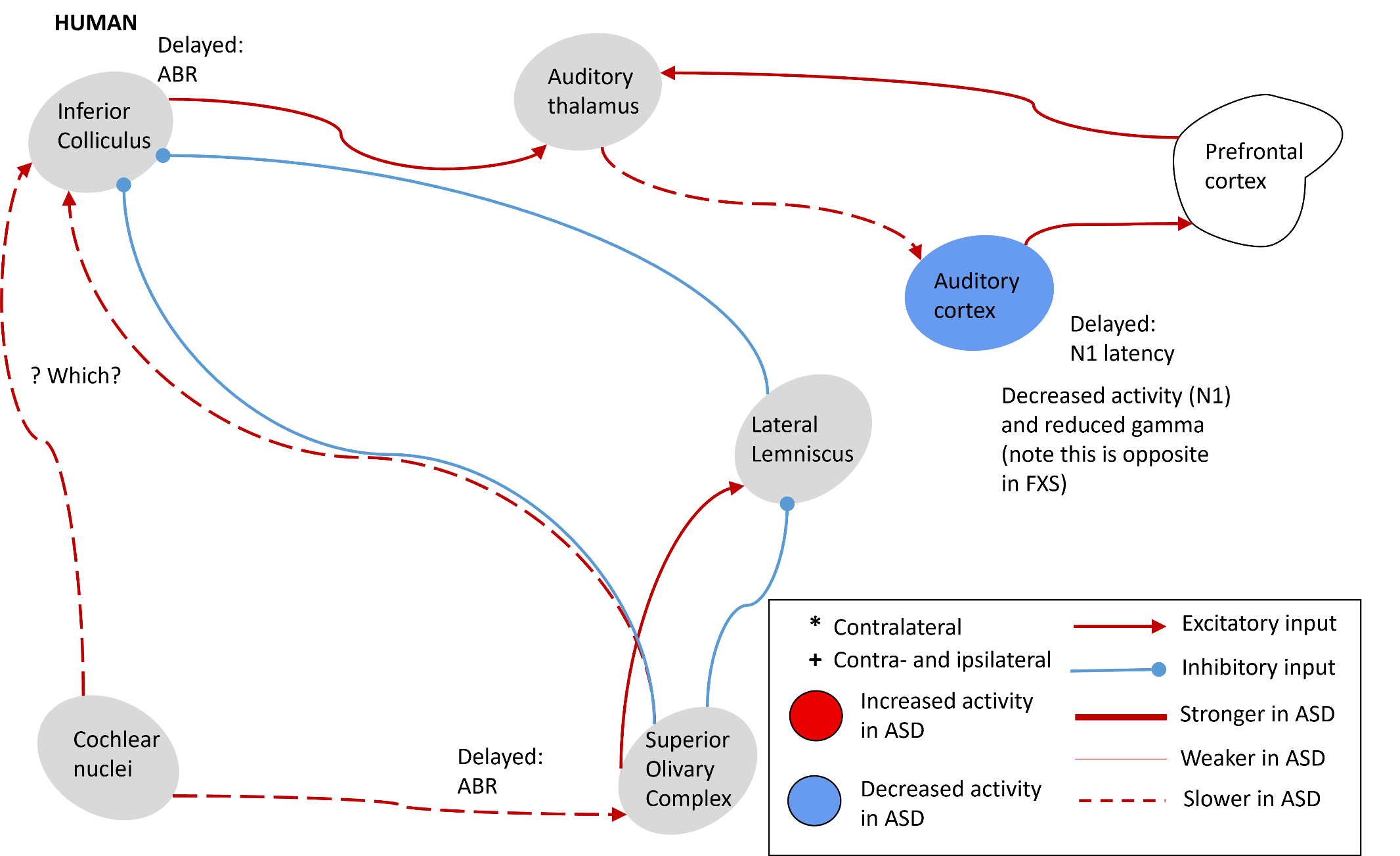

Supplement: Supplementary file 1 — Additional file 1: Supplementary Table 1. Primary research articles which passed the criteria for inclusion in the systematic review. Studies conducted using mouse models on the C57/Black6 background at an age over 3 months are indicated by an asterisk after the author name. Supplementary Table 2. Rodent models with auditory phenotypes described in this review. Gene names are formatted as in the cited papers, with alternate names for equivalent human genes used in SFARI gene in brackets where relevant. Supplementary Figure 1. The ascending auditory processing pathway with presumed changes in autism based on results from human studies. Excitatory (red) and inhibitory (blue) connections between structures along the pathway are illustrated along with the changes to these connections and the activity within areas in human autism studies. Connections are primarily ipsilateral unless otherwise stated. Responses in the superior olivary complex occur later, likely due to slower transmission from the cochlear nuclei. Responses in the inferior colliculus also occur later, but it is unclear whether it is input from the cochlear nuclei or the superior olivary complex, or both, which is delayed. Signals from the auditory thalamus take longer to reach the auditory cortex in human autism papers. There is decreased activity and increased latency (N1), and reduced gamma in the auditory cortex in human autism studies. [file 11689_2022_9458_MOESM1_ESM.docx]
